# Expert consensus on the management of chronic lymphocytic leukaemia in Asia

**DOI:** 10.1007/s10238-023-01007-2

**Published:** 2023-02-16

**Authors:** Eric Tse, Yok Lam Kwong, Yeow Tee Goh, Ping Chong Bee, Soo Chin Ng, Daryl Tan, Priscilla Caguioa, Huynh Nghia, Teresita Dumagay, Lalita Norasetthada, Suporn Chuncharunee, Vivek Radhakrishnan, Bhausaheb Bagal, Tubagus Djumhana Atmakusuma, Nadia Ayu Mulansari

**Affiliations:** 1 Division of Haematology, Medical Oncology and Haematopoietic Stem Cell Transplant, Department of Medicine, School of Clinical Medicine, The University of Hong Kong, Pok Fu Lam, Hong Kong China; 2 2.Division of Haematology, Medical Oncology and Haematopoietic Stem Cell Transplant, Department of Medicine, School of Clinical Medicine, The University of Hong Kong, Pok Fu Lam, Hong Kong China; 3 Department of Haematology, Singapore General Hospital, Singapore, Singapore; 4 Faculty of Medicine, University of Malaya, Kuala Lumpur, Malaysia; 5 Subang Jaya Medical Centre (SJMC), Selangor, Malaysia; 6 Mount Elizabeth Novena Hospital, Singapore, Singapore; 7 Section of Haematology, St Luke’s Medical Center, University of Santo Tomas Hospital, Manila, Philippines; 8 Blood Transfusion and Haematology Hospital (BTH), Ho Chi Minh, Vietnam; 9 Division of Haematology, Department of Medicine, Philippine General Hospital, Manila, Philippines; 10 Division of Haematology, Department of Internal Medicine, Faculty of Medicine, Chiang Mai University, Chiang Mai, Thailand; 11 Department of Medicine, Ramathibodi Hospital, Mahidol University, Bangkok, Thailand; 12 Clinical Haematology Oncology and HCT, Tata Medical Centre, Kolkata, India; 13 Department of Medical Oncology, Tata Memorial Centre, Parel, India; 14 Haematology-Medical Oncology Division, Dr. Cipto Mangunkusumo National General Hospital/ Universitas Indonesia, Jakarta, Indonesia

**Keywords:** Chronic lymphocytic leukaemia, BTK inhibitors, Bcl-2 inhibitors, Treatment, Asia

## Abstract

In recent years, considerable progress has been made in the standard treatment for chronic lymphocytic leukaemia (CLL) due to the availability of new potent drugs. However, the majority of data on CLL were derived from Western populations, with limited studies and guidelines on the management of CLL from an Asian population perspective. This consensus guideline aims to understand treatment challenges and suggest appropriate management approaches for CLL in the Asian population and other countries with a similar socio-economic profile. The following recommendations are based on a consensus by experts and an extensive literature review and contribute towards uniform patient care in Asia.

## Introduction

Chronic lymphocytic leukaemia (CLL) is a clonal mature B-cell neoplasm characterised by lymphocytosis of B-cells with a distinct immunophenotype [1]. It is the most common type of leukaemia in adults and accounts for 30% of all leukaemia cases in Western countries [2, 3]. The annual incidence rate of CLL is approximately 4.92 and 2–6 per 100,000 persons per year in Europe and the USA, respectively [2, 4]. The median age of patients at the time of diagnosis is 71 years, and > 95% of CLL patients are over 50 years old [2].

The incidence of CLL is less frequent in individuals of Asian and Middle Eastern ancestry [2, 4]. The reported age-adjusted incidence of Asian or Pacific Islander males is only 1.5 per 100,000 persons [4]. This low incidence of CLL persists even among Asian migrants and their descendants in western countries [5]. In contrast, a surveillance study conducted by Clarke et al. reported an increase in the incidence of CLL among the Asian population living in the USA, suggesting a strong impact of environmental factors, which changes as a result of immigration and acculturation, on the disease aetiology [6]. In the past decades, data on CLL were mostly derived from Western countries, with limited studies conducted on Asian populations [4, 5]. The specific causes for the low incidence of CLL in the Asian population are unknown; however, clinical-epidemiologic investigations may provide insights into it.

Although significant efforts have been made to improve the management of CLL patients in Asia, there is a dire need for a consensus document that can guide physicians in the diagnosis and treatment of CLL. Currently, there are no guidelines on the management of CLL from an Asian perspective [5]. The present document will be instrumental in understanding the current CLL treatment scenario across Asia, identifying the treatment challenges, and suggesting appropriate management approaches for CLL in the Asian population and other countries with a similar socio-economic profile. This document has been developed based on a consensus by experts, which was formulated after an extensive literature review of the available evidence. However, the final decision regarding the care of a patient with CLL should be made by the treating physician after considering patient-specific factors.

## Methodology

A consensus statement was developed, using a modified Delphi method, among a panel of 15 haematology oncology experts. To identify the scope of this consensus statement, the literature on CLL management was first reviewed on MEDLINE (via PubMed) database. A thorough literature search was conducted to identify articles written in English published between 1 January 2010 and 31 April 2021. The keywords related to CLL were paired with terms such as diagnosis, risk stratification, treatment, prognosis, Bruton’s tyrosine kinase inhibitor (BTKi), B-cell lymphoma-2 inhibitors (Bcl-2i), TP53, and immunoglobulin heavy chain variable region gene (IGHV) mutation to identify relevant articles. The results of the literature searches were used to develop a qualitative survey. Panel members were asked to complete one round of surveys via email. The survey was divided into three sections: diagnosis, prognosis, and treatment of CLL. A total of 40 questions were presented, and their responses were obtained. The survey was followed by a meeting on 2 October 2021, during which the results were presented and statements were discussed.

The statements were categorised as follows:

- Consensus: Statements achieving a score of 70% or higher
- Near consensus: Statements achieving a score of 65–69%
- No consensus: Statements that did not meet the criteria of consensus or near consensus

Statements with near consensus or no consensus were discussed after each round to evaluate if they should be refined and added to the following Delphi survey or omitted completely. This methodology did not require approval from the ethics committee.

**Fig. 1:**
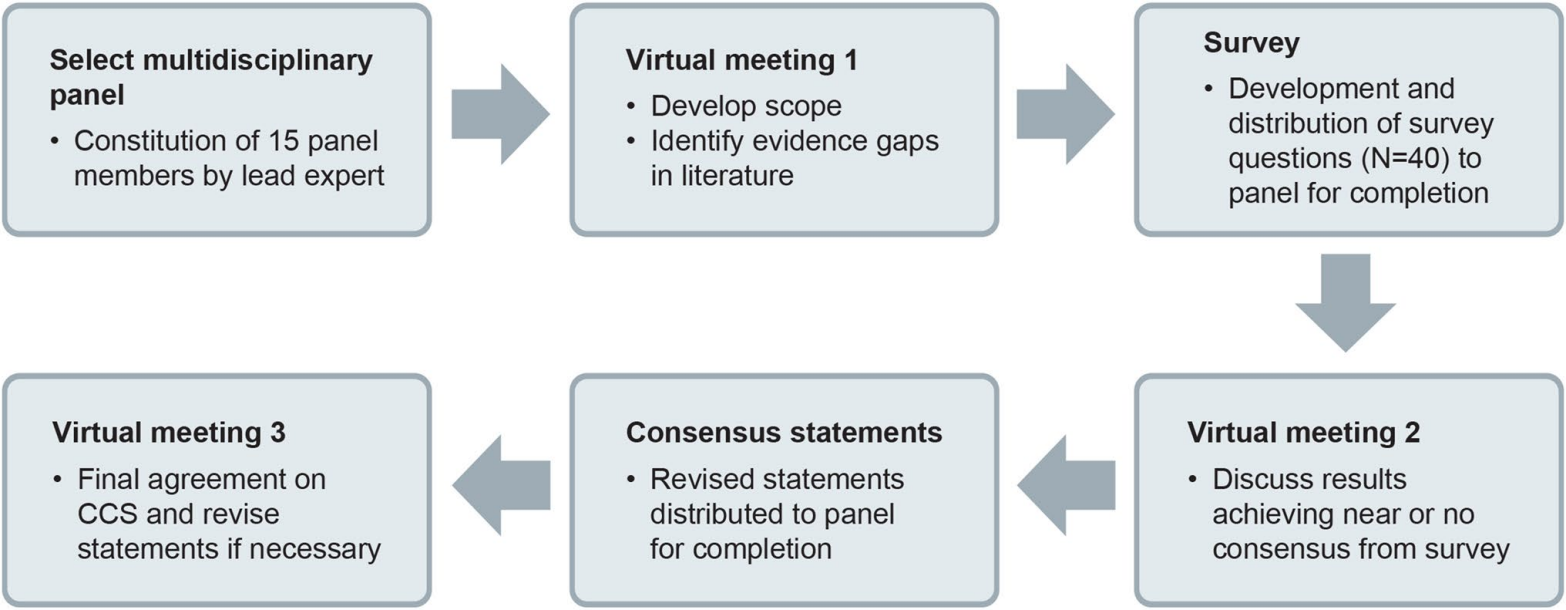
Consensus process using a modified Delphi method

Figure 1 provides an overview of the consensus process used to create the clinical consensus statement (CCS).

Thirty-five consensus recommendations were made at the end of the CCS process, which were based on thorough discussion and available evidence. A summary of key recommendations and the mean CCS score is provided in Table 1. The consensus recommendations and the supporting literature will be discussed in subsequent sections.

**Table 1:** Summary of key consensus statements for chronic lymphocytic leukaemia management

	Statements	Mean CCS score (%)
*Diagnosis*		
1	An amount of circulating monoclonal B lymphocytes cells ≥ 5 × 10^9^/L in peripheral blood (PB) for more than three months, which express CD19, CD5, and CD23, is sufficient to diagnose chronic lymphocytic leukaemia (CLL)	73
2	Complete blood count and flow cytometry of peripheral blood (PB) with immunophenotyping using cell surface markers are adequate for the diagnosis of CLL	89
3	Bone marrow biopsy and aspiration are not required for routine CLL diagnosis and should only be performed if the patient exhibits thrombocytopenia, cytopenia, anaemia, and/or small lymphocytic leukaemia (SLL) diagnosis is suspected	67
4	Imaging techniques, such as ultrasound, computed tomography, magnetic resonance imaging, and positron emission tomography-CT (PET-CT) scanning are important to identify index nodes for biopsy when transformation is suspected and to also locate enlarged nodes that are giving compression symptoms to the patients but are not recommended for routine CLL diagnosis or staging due to financial constraints (cost consideration) of patients	53
*Prognosis*		
5	Both Rai and Binet clinical staging systems are recommended for use in CLL	73
6	Perform FISH for del17p and test for TP53 aberrations before initiating first-line treatment and/or each line of treatment for relapsed/refractory (r/r) CLL patients	80
7	Testing for serum beta-2 microglobulin (s-β2M), del13q, and del11q is recommended in patients with previously untreated CLL	80
8	IGHV mutational test has a prognostic value, but it may not be mandatory to determine the choice of treatment. Performing IGHV mutational test is also dependent on the age of the patient—older patients (> 75 years) may not necessarily require it	67
9	If the patient is well-insured/able to afford it, it is highly recommended that both FISH for del17p and test for TP53 be done simultaneously as relying on only del17p alone may risk missing TP53 aberrations. However, if cost is an issue, patients may only be tested for TP53 aberrations if they test negative for del17p	67
10	The CLL-IPI scoring system is only validated for patients treated with chemoimmunotherapy (CIT). This scoring system would not be needed/routinely used for patients on novel agents for treatment	53
11	There is no one-size-fits-all fitness assessment technique that has been proven to be the best for deciding on CLL therapy, but it should include disease stage, cytogenetic risk, and the patient’s physical condition and comorbidities	53
*Treatment*		
12	A watchful waiting approach is recommended for newly diagnosed patients with asymptomatic early stage disease (Binet A–B, Rai 0–ii) unless they have evidence of disease progression	93
13	iwCLL guidelines should be followed before initiating treatment for all treatment-naïve and r/r CLL patients. Necessary indications (according to iwCLL criteria) for treatment initiation include: Evidence of progressive bone marrow failure manifested by the development of anaemia and/or thrombocytopenia (cut-off levels of Hb < 10 g/dL or platelet counts < 100 × 10^9^/L) Massive splenomegaly, which is progressive or symptomatic (i.e. ≥ 6 cm below the left costal margin) Massive lymph node or a progressive or symptomatic lymph node (i.e. ≥ 10 cm in longest diameter) Progressive lymphocytosis with an increase of ≥ 50% within 2 months or lymphocyte doubling time (LDT) < 6 months Autoimmune disease (anaemia and/or thrombocytopenia) with poor response to corticosteroids or other standard treatments Symptomatic or functional extra-nodal involvement (e.g. skin, kidney, lung, spine) Disease-related symptoms, which are defined as any of the following: unintentional weight loss of ≥ 10% in the past 6 months, significant fatigue, fever higher than 38^0^C for ≥ 2 weeks without evidence of infection, and night sweats for > 1 month without evidence of infection	93
14	CIT may still remain a useful option for first-line treatment of choice for young and fit patients with IGHV mutation	100
15	BTKi is the preferred first-line treatment of choice for patients with del17p or TP53 mutation	80
16	BTKi can be considered in all CLL patients requiring therapy, including those with high-risk genomic features, such as TP53 abnormalities	93
17	Patients who are intolerant to ibrutinib or who have relative contraindications to ibrutinib may still tolerate acalabrutinib	73
18	Second-generation BTKi including acalabrutinib may have a better safety profile than ibrutinib, especially in patients with high-risk disease characteristics	93
19	Bcl-2i should be placed after BTKi in r/r CLL	80
20	BTKi followed by Bcl-2i is the recommended sequencing strategy while using novel therapies in CLL	80
21	Bcl-2i can be considered in all CLL patients in need of therapy, including those with high-risk genomic features such as TP53 abnormalities	73
22	Measurable residual disease (MRD) evaluation may be considered after 6 cycles of therapy regimens	73
23	BTKi may be considered the preferred first-line treatment of choice for patients without del17p or TP53 mutation and unmutated IGHV (IGHV-u), > 65 years of age or with significant comorbidities	67
24	BTKi may be considered the preferred r/r therapy of choice for patients WITH del17p or TP53 mutation	67
25	Preferred first-line treatment of choice for young and fit patients with IGHV-u needs to be validated further	47
26	Preferred first-line treatment of choice for “young and fit” and “frail” patients with comorbidities needs to be validated further	57
27	Preferred first-line and r/r treatment of choice for patients without del17p or TP53 mutation and IGHV-u and who are < 65 years of age need to be validated further	53
28	Preferred first-line and r/r treatment of choice for patients without del17p or TP53 mutation and mutated IGHV (IGHV-m) need to be validated further	51
29	More evidence is required on the changes in the treatment of choice in first-line or r/r settings in the absence of immunogenetic testing (IGHV mutational status unknown)	60
30	More evidence is required to confirm whether CIT can be considered in all CLL patients in need of therapy, including those with high-risk genomic features, such as TP53 abnormalities and IGHV-u	60
31	Further validation is required for the antibody panel measured for MRD assessment according to the European Research Initiative on CLL (ERIC) 4- or 6-colour protocols	47
32	More evidence is required to consider allogeneic stem cell transplantation only for patients after they have become unresponsive to other therapies	27

## Diagnosis

In patients suspected of having CLL, it is critical to evaluate the blood smear, immunophenotype, and genetic features of circulating lymphoid cells to rule out other lymphoproliferative diseases that can be misdiagnosed as CLL [7]. The CLL diagnosis and treatment guidelines have recently been updated [7]. Diagnosis of CLL is defined by the presence of ≥ 5 × 10^9^/L monoclonal B lymphocytes in peripheral blood (PB) for more than three months, with an expression of CD19, CD5, and CD23 [2, 7]. Immunophenotyping of blood lymphocytes is an important step in assessing clonality and determining the number of CD19^+^ CD5^+^ B lymphocytes [8]. The detection of immunoglobulin light chain restriction using flow cytometry can confirm the clonality of these B lymphocytes [7].

Lymph node infiltration by small lymphocytes with a CLL phenotype in the absence of lymphocytosis ≥ 5 × 10^9^/L leads to the diagnosis of small lymphocytic lymphoma (SLL). The diagnosis of monoclonal B lymphocytosis (MBL) should be made in the presence of a clone at a level of < 5 × 10^9^/L with an immunophenotypic profile identical to that observed in CLL and an absence of bone marrow failure or peripheral lymphadenopathy [8].

The typical characteristic profile exhibited by CLL lymphocytes include CD19 + , CD5 + , CD23 + , CD20 + low, CD200 + , CD22 + low/negative, CD79b + low/negative, CD43 + low, sIgκ + or sIgλ + low, sIgM + low, CD11c + low/negative, FMC7-, CD10-, and CD103- [7]. Lymphocytes in CLL co-express the surface antigen CD5 with the B-cell antigens CD19^+^, CD20^+^, and CD23^+^. The levels of surface immunoglobulin CD20^+^ and CD79b^+^ are characteristically low compared with those found on normal B-cells. A large harmonisation effort by Rawstron et al. [9] demonstrated that a panel of CD19^+^, CD5^+^, CD20^+^, CD23^+^, k, and λ markers is sufficient for the diagnosis of CLL. However, a subset of CLL, including those with trisomy 12, can often express high levels of key markers including surface IgM and CD20 and diagnosis should remain if the clonal B-cells are CD5 + and CD23 + [10]. Genomic and molecular testing is not widely available or established in certain regions in Asia (Tables 2 and 3).

### Recommendations

- The presence of circulating monoclonal B lymphocytes ≥ 5 × 10^9^/L with the characteristic immunophenotype in PB for more than three months is sufficient to diagnose CLL.
- Imaging techniques, such as ultrasound, computed tomography (CT), magnetic resonance imaging (MRI), and positron emission tomography-CT (PET-CT) scanning are important to identify index nodes for biopsy when transformation is suspected and to locate enlarged nodes that cause symptoms in patients, but are not recommended for routine CLL diagnosis or staging.
- Bone marrow biopsy and aspiration are not essential for routine CLL diagnosis and should only be conducted if the patient exhibits thrombocytopenia, cytopenia, anaemia, and/or small lymphocytic leukaemia (SLL) diagnosis is suspected.
- Although genomic and molecular tests are not required to diagnose CLL, these tests may help predict the prognosis.

## Prognosis

### Clinical staging

Rai and Binet are two widely accepted staging systems used in both patient care and clinical trials. Both systems describe three major prognostic groups with distinct clinical outcomes and can be applied by physicians all over the world in clinical practice Table 2 [2, 7]. Both systems take into account lymph node, spleen, and liver involvement, as well as the presence of cytopenia due to marrow infiltration and, do not require imaging studies [2, 7]. Rai and Binet’s clinical staging systems are unable to predict an individual patient’s ongoing clinical course, particularly in the early stages [11]. As a result, other prognostic biomarkers have been identified in an attempt to predict disease progression [2, 11].

**Table 2:** Clinical staging systems and risk status

Risk status	Modified Rai stage	Binet stage
Low risk	0: Lymphocytosis	A: < 3 involved nodal areas
Intermediate risk	I: Lymphadenopathy	B: ≥ 3 involved nodal areas
	II: Splenomegaly and/or hepatomegaly	
High risk	III: Haemoglobin < 11 g/dL	C: Haemoglobin < 10 g/dL and/or platelets < 100 × 10^9^/L

### Prognostic biomarkers

Over the decades, a greater understanding of prognostic indicators, such as serum markers, flow cytometry results, immunoglobulin heavy chain variable region gene (IGHV) mutation status, microRNAs, chromosomal anomalies, and gene mutations, has aided prognosis in CLL [12]. The most relevant prognostic parameters identified Table 3 to date are IGHV mutational status, serum markers, presence of chromosome anomalies (del13q, del11q, tri12, del17p), and gene mutations (*TP53* mutations) [7].

**Table 3:** Key chronic lymphocytic leukaemia prognostic biomarkers discussed [12]

Category	Prognostic markers
Serum markers	s-β2M, thymidine kinase (s-TK), lactic dehydrogenase (LDH)
IGHV mutation status	Mutated IGHV (IGHV-m), unmutated IGHV (IGHV-u)
Chromosome anomalies	del13q, del11q, del17p, tri12
Gene mutations	*TP53*

#### Serum markers

Serological tests are inexpensive and play an important role in the diagnosis and prognostic evaluation of CLL. The most common standard serum markers used to predict poor outcomes are beta 2-microglobulin (β2M), thymidine kinase (TK), and lactic dehydrogenase (LDH) [12].

Lymphocyte doubling time (LDT) is a simple parameter with a distinct prognostic significance in the clinical management of CLL patients and has been used for more than three decades [12, 13]. Patients with an LDT of ≤ 12 months are associated with poor prognosis, whereas an LDT > 12 months is indicative of good prognosis, as reflected by a long treatment-free period and survival [13]. β2M and TK levels provide independent prognostic information on progression-free survival (PFS) in CLL [14]. Elevated β2M levels are associated with a poor prognosis and are widely used to improve risk stratification [12, 14]. A high TK level, which correlates with a shorter LDT and IGHV unmutated status, indicates a high risk of disease progression [15]. The LDH level is an indicator of time-to-first treatment (TTFT), and a high level is associated with PFS, overall survival (OS), and Richter’s transformation [12, 16].

#### IGHV mutation status

IGHV mutation status plays a key role in the prognosis of CLL. Based on the sequence identity of germline *IGHV* [17, 18], CLL clones can be classified into: *IGHV*-mutated (*IGHV*-m), in case more than 2% of the *IGHV* DNA sequence is mutated; and *IGHV* unmutated (*IGHV*-u) in case there are less than 2% of somatic hypermutations in *IGHV* [2, 18]. In treatment-naive patients, the *IGHV*-u status, which correlates to quicker LDT and CD38 overexpression, predicts shorter TTFT as it is linked to a more aggressive course of CLL, whereas M-CLL patients exhibit a better prognosis [10, 12, 18]. However, the use of IGHV mutation status in clinical practice is limited in resource-restrained countries as it involves expensive molecular techniques [10]. The majority of Asian countries do not have *IGHV* mutational status testing facilities available, and this may pose challenges when it comes to treatment decisions and prognosis.

#### Chromosome aberrations

In recent years, fluorescence in situ hybridisation (FISH), karyotyping, and next-generation sequencing have all been extensively used in the diagnosis and risk stratification of CLL, as well as in treatment decisions and clinical trial design [12]. FISH is sensitive in detecting major chromosomal abnormalities typically identified in CLL.

Based on the data from the Surveillance, Epidemiology, and End Results (SEER) Patterns of Care (POC), Seymour et al. [19] compared testing and treatment patterns of CLL patients who were diagnosed in 2008 (*n* = 1008) with patients diagnosed in 2014 (*n* = 1367) in a real-world study. The most prevalent chromosomal aberrations discovered were deletions of the long arm of chromosome 13 (del13q), trisomy 12 (tri12), deletions of the long arm of chromosome 11 (del11q), and deletions of the short arm of chromosome 17 (del17p) [12, 19].

Deletion of 13q, specifically involving band 13q14, is the most common cytogenetic aberration in CLL and isolated del13q is associated with the longest survival [12, 20]. Given that 13q deletions in CLL are heterogeneous in size, the del 13q14 region, which is implicated in the biology and clinical characterisation of CLL, is likely to contain more than one tumour suppressor gene [20]. On the other hand, patients with 60% or more CLL cells with del(13q) have shorter treatment-free survival (TFS) and OS [12, 21].^8^ Deletion of 11q is observed in 8–19% of CLL cases [12, 20]. Patients with CLL and del11q experience a more aggressive disease course characterised by extensive nodal disease, and shorter PFS and TFS [22]. In approximately 5–8% of cases, del(17p) is detected at initial diagnosis; it is associated with a poor prognosis and drives first-line treatment decisions [2, 12]. As a result, assessment for 17p deletion by FISH is mandatory for determining therapeutic approaches [8]. A better description of the clinical significance of these chromosomal abnormalities is required.

Recent data by Baliakas et al*.* [23] suggest that complex karyotype (CK) defined by the existence of ≥ 3 chromosomal abnormalities detected by conventional karyotyping may have an important prognostic value for treatment decision-making in CLL. According to the study, patients with ≥ 5 abnormalities (high-CK) exhibit poor clinical outcomes independent of clinical stage, TP53 status, or IGHV status. Cases with 3–4 aberrations (low-CK and intermediate-CK, respectively) are clinically relevant only in the presence of TP53 aberrations. On the contrary, patients with CK and trisomies of chromosomes 12 and 19 displayed an excellent prognosis with favourable outcomes and the longest OS [23, 24]. Clinical validation is certainly required before utilising prognostic models involving CK in the risk stratification of CLL in practice [23].

#### Gene mutations

Next-generation sequencing (NGS) analysis can be utilised for routine gene mutation screening in CLL as it is a sensitive, reproducible, and resource-efficient tool [25]. The presence of mutations by NGS was associated with IGHV-u status, CD38 expression, and complex karyotypes [12, 25]. In recent years, 44 recurrently mutated genes and 11 recurrent somatic copy number variations that drive CLL has been identified through whole-exome sequencing of 538 CLL and matched germline DNA samples [12, 26].

*TP53* gene, which encodes a transcription factor involved in cell cycle regulation and apoptosis, is located on chromosome 17p13.1 [20]. The *TP53* gene mutation or deletion is present in approximately 10% of treatment-naïve CLL cases and is associated with an unfavourable prognosis [2, 20]. CLL with *TP53* mutations may or may not have concomitant del17p [2]. It has been reported that patients with CLL harbouring del17p and *TP53* mutations without del17p have comparable PFS and OS [27]. As a result, the presence of either del17p or *TP53* mutations has prognostic significance and should be employed to guide therapeutic decisions [7, 27].

With the different prognostic parameters, numerous prognostic scoring and stratification systems based on multivariate analyses have been developed [7]. The CLL international prognostic index (CLL-IPI) is a relatively easy-to–use prognostic score that combines five parameters (clinical stage, age, IGHV mutational status, β2M level, and the presence of del17p and/or *TP53* mutations) to predict survival and TTFT in CLL patients treated with chemoimmunotherapy [28]. However, with the introduction and application of novel therapies, the impact of some of these prognostic markers such as del11q and IGHV-u becomes less significant [7].

#### Fitness of the patient

Assessment of the physical fitness of patients is an important parameter in treatment decisions, especially for elderly CLL patients. However, no specific fitness assessment tool has proved optimal for decision-making on CLL treatment as no two patients are comparable with regard to comorbidity burden, fitness/frailty, and physiologic function [29]. In general, the assessment should not only consider the disease stage and cytogenetic risk, but also the patient’s physical state and comorbidities. Comorbidities can be assessed by measures including the Cumulative Index Rating Scale (CIRS) and the Charlson Comorbidity Index (CCI) [2, 7, 29]. In clinical trials, patients with a CIRS score ≤ 6 and a normal renal function (creatinine clearance > 70 mL/min/1.73 m^2^) are considered “fit” for more intensive treatments [2, 29].

### Recommendations

- Both Rai and Binet clinical staging systems are recommended for use in CLL.
- Perform FISH for del17p and test for *TP53* gene mutations before initiating first-line treatment and each line of treatment for relapsed/refractory (r/r) CLL patients.
- If resources and accessibility permit, it is highly recommended that both FISH for del17p and test for TP53 gene mutations be done simultaneously, as relying on del17p alone is associated with the risk of missing *TP53* aberrations.
- IGHV mutational test has a prognostic value; however, its significance is less apparent in patients treated with novel therapeutic agents.
- Testing for β2M, del13q, and del11q is recommended in patients with previously untreated CLL.
- The CLL-IPI scoring system has only been validated for patients treated with chemoimmunotherapy (CIT).
- There is no one-size-fits-all fitness assessment technique that has been proven to be the best for deciding on CLL therapy. However, it should include disease stage, cytogenetic risk, and the patient’s physical condition and comorbidities.

## Treatment

### Indications for treatment

Not all patients with CLL require treatment at the time of diagnosis [30]. According to existing international guidelines, asymptomatic patients with early stage disease (Rai 0, Binet A) should be monitored without therapy unless there is evidence of disease development or disease-related symptoms [7, 11, 31]. Patients are usually monitored at intervals of 1–3 months with this wait-and-watch approach. Several studies have discovered that treating patients with early stage disease does not improve their survival outcomes [7, 30].

Treatment should be initiated when patients develop active disease according to the iwCLL criteria defined as follows [7]:

1. Evidence of progressive bone marrow failure manifested by the development of anaemia and/or thrombocytopenia (Hb < 10 g/dL or platelet counts < 100 × 10^9^/L).
2. Massive splenomegaly that is progressive or symptomatic (i.e. ≥ 6 cm below the left costal margin).
3. Massive lymph node or a progressive or symptomatic lymph node (i.e. ≥ 10 cm in longest diameter).
4. Progressive lymphocytosis with an increase of ≥ 50% within 2 months or LDT < 6 months.
5. Autoimmune disease (anaemia and/or thrombocytopenia) with poor response to corticosteroids or other standard treatments.
6. Symptomatic or functional extra-nodal involvement (e.g. skin, kidney, lung, spine).
7. Disease-related symptoms, which are defined as any of the following: unintentional weight loss of ≥ 10% in the past 6 months, significant fatigue, fever higher than 38 °C for ≥ 2 weeks without evidence of infection, and night sweats for > 1 month without evidence of infection.

### Chemoimmunotherapy (CIT)

#### Fludarabine, cyclophosphamide, and rituximab

For more than a decade, the anti-CD20 monoclonal antibody rituximab, in combination with fludarabine, and cyclophosphamide have been the gold standard for young and fit patients [31]. In the CLL-8 phase III randomised controlled trial by the German CLL Study Group (GCLLSG), the combination of fludarabine, cyclophosphamide, and rituximab (FCR) as first-line CIT improved the PFS and OS of physically fit CLL patients (glomerular filtration rate > 70 mL/min and CIRS score < 6) [32]. At 3 years, significantly more patients were free of disease progression in the CIT group (FCR) compared to the chemotherapy group (FC) (65% vs. 45%; *p* < 0·0001) [32]. A long-term follow-up of almost 6 years confirmed the benefit of FCR over FC in terms of significant improvement in PFS and OS [33]. The median PFS were 56.8 and 32.9 months for the FCR and FC group, respectively (Hazard ratio (HR) 0.59; *p* < 0.001), whereas the median OS was not reached in the FCR group versus 86 months in the FC group. The majority of CLL patients with IGHV-m responded very well to FCR versus FC [33]. In comparison with the rest of the cytogenetics subgroups, patients with del17p, IGHV-u, and del11q demonstrated a shorter PFS, emphasising the need to investigate different therapeutic options [33, 34].

A similar observation was reported by Thompson et al. [35] in the follow-up of the phase II FCR study at MD Anderson Cancer Centre. At a median of 12.8 years, significantly higher FCR-treated CLL patients with IGHV-m achieved long-term PFS (53.9%) as compared to that of patients with IGHV-u (8.7%). The high rate of long-term PFS in patients with IGHV-m after FCR further validates the benefit of FCR in the IGVH-m group. The FCR regimen appears to have the potential to cure a fraction of IGVH-m young and fit patients without del17p or del11q mutations [33–35]. Owing to the inferior outcome of IGHV-u CLL patients, alternate therapies should be considered [35].

In terms of adverse events, the FCR regimen is associated with considerable toxic effects, mainly severe haematological toxicity in 56% of patients and severe infections in 25% of patients during treatment [32]. Prolonged neutropenia (17%–35%) [32, 33] and an elevated risk of secondary neoplasia were reported in the long-term follow-up of patients treated with FCR [33]. Recent trials such as the CLL-13 trial by GCLLSG [36] or the FLAIR trial [37] are challenging the current standard CIT, shifting the standard of care to targeted agents in selected patients.

#### Bendamustine–rituximab

If CIT is the treatment of choice for older patients with CLL, the bendamustine–rituximab (BR) regimen is preferred over the FCR regimen [31]. The international phase III CLL-10 study was the first trial to investigate the non-inferiority of CIT with BR compared with the standard front-line therapy of FCR regimen for patients with CLL [38]. The median PFS was 41.7 and 55.2 months in the BR and FCR groups, respectively. The trial failed to show the non-inferiority of BR, but a higher incidence of toxic effects was observed with FCR in patients > 65 years. A good efficacy with BR in this group may support the use of BR in fit elderly patients [34, 38].

#### Obinutuzumab–chlorambucil

Chlorambucil has been a tolerable and efficacious therapy option for frail and elderly CLL patients for decades, despite showing relatively low overall response rates (ORR) in earlier trials [31, 39]. Anti-CD20 antibodies, including rituximab and obinutuzumab, were then added to chlorambucil to improve the response rates [31]. The phase 3, randomised CLL-11 trial compared rituximab–chlorambucil (RC) with obinutuzumab–chlorambucil (OC) and chlorambucil monotherapy in 781 patients with previously untreated CLL and associated conditions [40]. The combination of an anti-CD20 antibody with chlorambucil resulted in better ORR and PFS in these patients, with a median PFS of 11.1 (chlorambucil alone), 16.3 (RC), and 26.7 (OC) months, respectively. A higher rate of complete responses (CR) (20.7% vs. 7.0%) and longer PFS was reported with obinutuzumab compared to rituximab when each was combined with chlorambucil [31, 40]. The OC treatment arm reported more frequent infusion-related reactions and neutropenia than the RC arm, but the risk of infection was not increased. However, currently, new, well-tolerated agents such as BTKi or Bcl-2 inhibitors have superseded chlorambucil with or without anti-CD20 antibody as the standard of therapy in elderly or frail CLL patients [31].

### Bcl-2 inhibitors

#### Venetoclax

Venetoclax is an orally administered, highly selective inhibitor of the anti-apoptotic protein Bcl-2, which is overexpressed in CLL cells [41]. Randomised clinical trials have demonstrated the efficacy of fixed-duration therapy with venetoclax in combination with an anti-CD20 antibody in first-line and r/r CLL. In the randomised, open-label, phase 3 MURANO study, patients with r/r CLL were given venetoclax in combination with rituximab (six cycles every 4 weeks) for up to 2 years (24 cycles) versus six cycles of BR [42]. The combination of rituximab and venetoclax led to a significantly higher PFS than that of the BR group (84.9% vs. 36.3%; *p* < 0.001) after a median follow-up period of 23.8 months. This benefit was evident in all high-risk subgroups, including patients with del17p and patients with IGHV-u. Furthermore, the high overall response rate (ORR) of 91.9% resulted in an outstanding 83.5% undetectable measurable residual disease (uMRD) rate in the peripheral blood [42]. The ability to achieve uMRD was linked to a higher chance of survival. The MURANO trial led to the approval of venetoclax in combination with rituximab as a second-line treatment for all CLL patients, independent of their del17p status, by the United States Food and Drug Administration (FDA) in 2018 [31].

Results from the recent CLL-14 trial allowed venetoclax to make its way into the first-line treatment landscape of CLL. The open-label, phase III CLL-14 trial evaluated the superiority of the one-year fixed duration of venetoclax–obinutuzumab to that of chlorambucil–obinutuzumab in elderly patients with previously untreated CLL and coexisting conditions (*n* = 432) [43]. At a median follow-up of 28.1 months, the combination of venetoclax and obinutuzumab resulted in an improved 24-month PFS (88.2% vs. 64.1%), which was also observed in patients with del17p, *TP53* mutation, or both, as well as in patients with IGHV-u. The recently presented 3-year follow-up showed a high ongoing rate of uMRD, in addition to improved PFS, for the combination of venetoclax–obinutuzumab vs. chlorambucil–obinutuzumab (47% vs. 7%) 18 months after treatment [44]. Overall, the toxicity profile of venetoclax–obinutuzumab remained similar to that of previous reports [44]. High response rates and deep remissions, and an extended PFS are seen in patients with venetoclax, thereby providing physicians with a time-limited therapeutic option [31, 44].

### BTK inhibitors

#### Ibrutinib

Ibrutinib is a first-in–class, oral, covalent inhibitor of Bruton’s tyrosine kinase (BTK) and inhibits CLL-cell migration, survival, and proliferation [31]. Ibrutinib was first evaluated in a phase III randomised controlled trial RESONATE-2 involving treatment-naive elderly CLL patients (non-del17pl, > 65 years), wherein its efficacy was compared with chlorambucil [45]. Ibrutinib was superior to chlorambucil as assessed by a 2-year PFS (89% vs. 34%), 2-year OS (98% vs. 85%), overall response rate (86% vs. 35%), and improvement in haematologic variables [45]. The long-term, 5-year follow-up reported a PFS and OS of 70% and 83%, respectively, for ibrutinib compared to 12% and 68%, respectively, for chlorambucil. Progression-free survival was also observed in patients with high-risk prognostic features, such as del11q or IGHV-u. The investigator-assessed ORR was 92%, with a CR rate of 30% [46]. This trial led to the approval for ibrutinib monotherapy in patients with previously untreated CLL [31].

With a median follow-up of 65.3 months, the RESONATE trial’s final analysis confirmed ibrutinib’s substantial effectiveness in patients with r/r CLL. Patients treated with ibrutinib and ofatumumab had median PFS of 44.1 and 8.1 months, respectively (*p* < 0.001). All high-risk categories of patients (del17p, TP53 mutation, IGHV-u status, del11q) showed similar results, accounting for 82% of the entire study population [47].

#### Acalabrutinib

Acalabrutinib is a second-generation, irreversible inhibitor of BTK with equipotent BTK inhibition as ibrutinib. It is associated with minimal off-target effects on other kinases [31, 34]. In the phase III, multicentre ELEVATE-TN study, the efficacy of acalabrutinib was evaluated in treatment-naïve CLL patients (CIRS score > 6 and creatinine clearance of < 70 mL/min) [48]. Patients were randomised to receive acalabrutinib (Acb) with (*n* = 179) or without (*n* = 179) obinutuzumab (G) or chlorambucil (Clb) with obinutuzumab (*n* = 177). When compared to obinutuzumab–chlorambucil, acalabrutinib alone and in combination with obinutuzumab reduced the risk of disease progression by 80% and 90%, respectively, after a median follow-up of 28.3 months. A similar benefit was seen across subgroups, including del17p, complex karyotype, and IGVH-u [48]. Acalabrutinib may be considered an option for elderly, unfit patients with high-risk diseases [34].

Acalabrutinib was compared to the investigator’s choice (idelalisib plus rituximab [IR] or bendamustine plus rituximab [BR]) in the phase III, multicentre ASCEND study in patients with r/r CLL [49]. In this study, 16% of the study population had a del17p with an estimated 12-month PFS of 88% for acalabrutinib vs. 68% or 69% for IR or BR, respectively. Acalabrutinib demonstrated a better safety profile when compared to IR, with serious adverse events occurring in 29% (acalabrutinib monotherapy), 56% (IR), and 26% (BR) of patients. Findings from this trial point to the use of acalabrutinib as a viable treatment option for patients with r/r CLL, including patients with high-risk disease characteristics [49].

### Combination of BTKi and Bcl-2i

With a fixed-duration approach, Bcl-2i venetoclax can achieve deep remissions, resulting in high rates of uMRD in treatment-naive and r/r CLL patients [31, 43, 44]. In contrast, BTKi such as ibrutinib and acalabrutinib seldom achieve a complete remission or uMRD status but still produce high ORR, long-term disease control, and survival benefits [50]. Ongoing studies are now evaluating the clinically complimentary activity of BTKi and Bcl-2i as combination therapies [31, 50]. The treatment combination of BTKi (ibrutinib) and Bcl-2i (venetoclax) in first-line [51] and r/r [52] treatment settings in clinical trials showed a high uMRD rate of 36% in r/r CLL patients [52] and 61% in untreated high-risk and older patients with CLL [51].

### First-line and relapsed/refractory treatment

Current guidelines recommend treatment with continuous BTKi or 1-year fixed duration of venetoclax and obinutuzumab combination for patients with del17p or TP53 mutation and/or IGHV-u. Chemoimmunotherapy (chlorambucil/obinutuzumab, bendamustine/rituximab or FCR) is still an available treatment option in treatment-naïve, IGHV-m CLL patients without del17p, TP53 mutation, or complex karyotype [31, 53, 54]. Novel therapeutics are superior to CIT regimens, leading to significantly better survival in r/r CLL patients [31, 53, 54]. The therapeutic agents recommended for CLL patients depend on various parameters such as age, functional status, presence of comorbidities (arterial hypertension or renal impairment), organ function, and patient preference.

### Allogeneic stem cell transplant

The indications for allogeneic hematopoietic cell transplantation (HCT) are limited due to the introduction of novel agents. Allogeneic HCT is now relegated to later stages of r/r CLL [55]. In young patients with high-risk diseases and limited availability of novel agents, allogeneic HCT may still be a feasible option [34].

### Recommendations

**Fig. 2:**
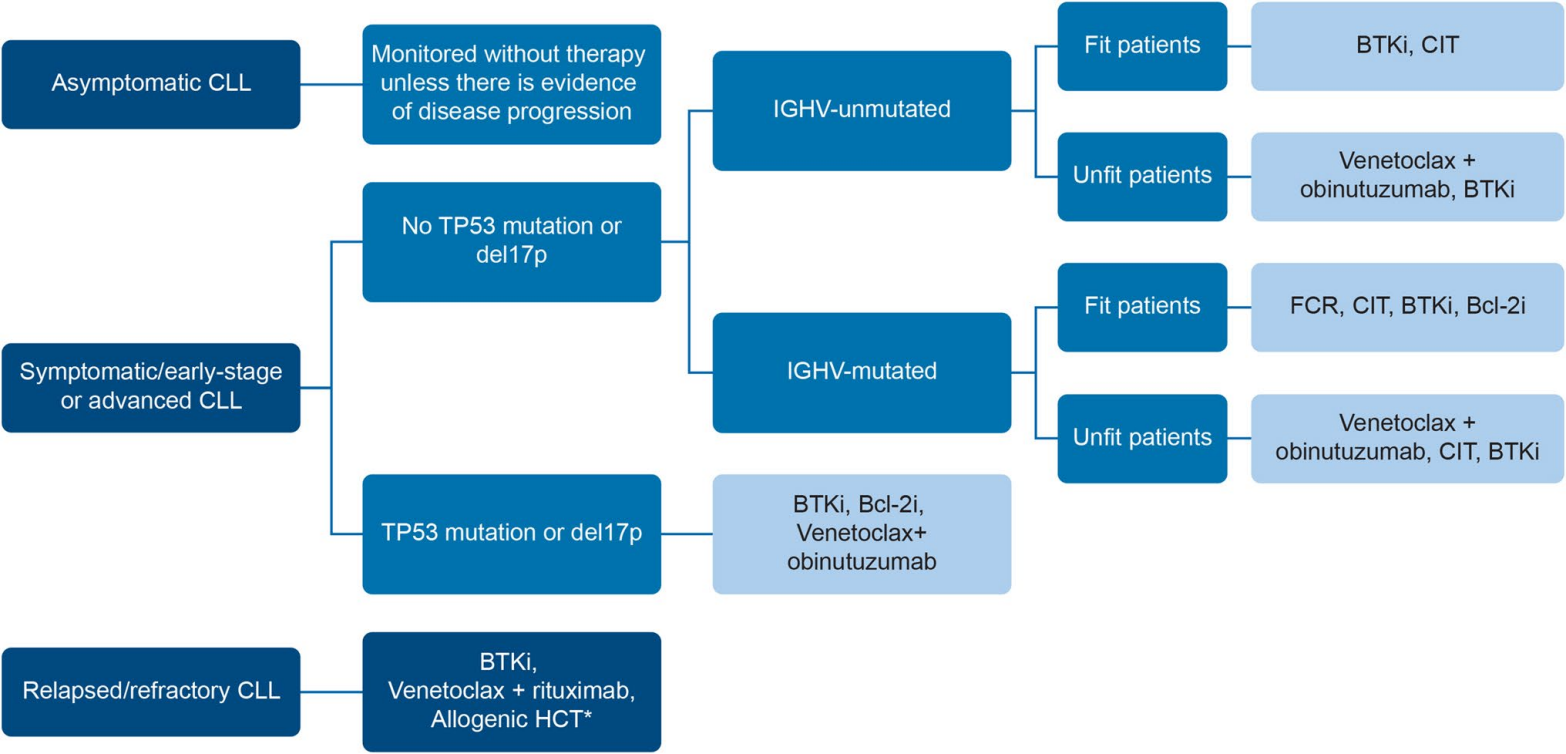
Flow diagram of the recommended treatment algorithm for CLL. *Allogenic HCT may be considered an option in patients who are young and have failed all existing therapies. *BTKi* Bruton’s tyrosine kinase inhibitor, *Bcl-2i* B-cell lymphoma-2 inhibitors, *CLL* chronic lymphocytic leukaemia, *CIT* chemoimmunotherapy, *HCT* hematopoietic cell transplantation, *IGHV* immunoglobulin heavy chain variable region gene

- The International Workshop on CLL (iwCLL) guidelines should be used for both the indication for treatment and evaluation of therapy.
- Asymptomatic patients with early stage disease (Rai 0, Binet A) should be monitored without therapy unless there is evidence of disease development or disease-related symptoms (Fig. 2).
- First-line treatment:
- In fit patients < 65 years of age with IGHV-m and CIRS < 6, either FCR or other novel agents may be considered (Fig. 2). Physicians need to inform young patients about the risk of secondary malignancy and offer the option of CIT or novel agents (BTKi) as an equivalent alternative.
- Patients with del17p or TP53 mutation should be offered BTKi as first-line treatment because of the demonstrated high response rates and potentially long-lasting remissions in this high-risk population. Fixed duration of venetoclax + obinutuzumab may also be considered in this clinical setting.
- In patients with significant comorbidities without del17p or TP53 mutations and IGHV-u, BTKi is a reasonable treatment option and could be used in preference to CIT because of lower toxicity and better efficacy. However, due to cost considerations and difficulty to obtain approval for the use of novel agents, CIT can be used as a first-line treatment option in this group of patients.
- Both BTKi and Bcl-2i have good clinical data to support their use in patients without del17p or TP53 mutation and IGHV-m. The advantage of Bcl-2i in this group is the finite treatment duration (1 year).
- Relapsed/refractory
- BTKi or venetoclax in combination with rituximab may be considered the preferred treatment of choice for patients with r/r CLL irrespective of del17p, TP53 mutations, and IGHV mutation status (Fig. 2).
- BTKi and Bcl-2i can be considered in all CLL patients in need of therapy, including those with high-risk genomic features, such as TP53 abnormalities.
- Acalabrutinib may be tolerated by patients who are intolerant to ibrutinib or have relative contraindications to ibrutinib. Acalabrutinib has a better safety profile than ibrutinib. If a patient has cardiovascular problems or high bleeding risk, acalabrutinib is preferred over ibrutinib.
- Bcl-2i after BTKi is the preferred sequence of drugs for the treatment of CLL as validated by evidence. Data on the patient response from treatment involving Bcl-2i followed by a BTKi is not robust.
- Measurable residual disease (MRD) evaluation may be considered at the end of treatment after CIT or the venetoclax + anti-CD 20 antibody combination to predict the risk of relapse and overall prognosis. MRD evaluation is available in Singapore, Malaysia, Thailand, India, and Hong Kong. MRD evaluation is not available in Vietnam and the Philippines.
- Older patients are not usually considered for allogeneic HCT due to the high mortality rate. Allogenic HCT may be considered an option in patients who are young and have failed all existing therapies.

## Conclusion

Chemoimmunotherapy plays a viable role in the treatment landscape of CLL patients in Asia with oral inhibitors reserved for high-risk CLL patients and/or in elderly patients who are less likely to tolerate chemotherapy regimens. BTKis and Bcl-2is are oral agents with excellent efficacy and are associated with different toxicity profiles. However, due to economic issues surrounding the treatment of CLL with novel therapeutic agents in Asia, the ability to prescribe these small molecules is limited. The goal for this new decade is to build on the early success of novel therapies by developing treatment regimens that reduce medication exposure time, risks of adverse effects, resistance, as well as treatment costs, which may increase the probability of reimbursement in these regions.
